# Towards the Development of a Novel Ex Ovo Model of Infection to Pre-Screen Biomaterials Intended for Treating Chronic Wounds

**DOI:** 10.3390/jfb11020037

**Published:** 2020-06-02

**Authors:** Elena García-Gareta, Justyna Binkowska, Nupur Kohli, Vaibhav Sharma

**Affiliations:** 1 Regenerative Biomaterials Group, The RAFT Institute & The Griffin Institute, Northwick Park & Saint Mark’s Hospitals, Harrow, London HA1 3UJ, UK; j.m.binkowska@gmail.com (J.B.); n.kohli@imperial.ac.uk (N.K.); sharmav@raft.ac.uk (V.S.); 2 Division of Biomaterials and Tissue Engineering, Eastman Dental Institute, University College London, London WC1X 8LD, UK; 3 Biomechanics Research Group, Department of Mechanical Engineering, Imperial Collage London, South Kensington Campus, London SW7 2AZ, UK

**Keywords:** chronic wounds, infection, chick embryo CAM, ex ovo, biomaterials

## Abstract

This communication reports preliminary data towards the development of a live ex vivo model of persistent infection that is based on the chick embryo chorioallantoic membrane (CAM), which can be used for pre-screening biomaterials with antimicrobial properties for their antimicrobial and angiogenic potential. Our results showed that it was possible to infect chicken embryos with *Staphylococcus aureus*, one of the main types of bacteria found in the persistent infection associated with chronic wounds, and maintain the embryos’ survival for up to 48 h. Survival of the embryos varied with the dose of bacteria inoculum and with the use and time of streptomycin application after infection. In infected yet viable embryos, the blood vessels network of the CAM was maintained with minimal disruption. Microbiological tests could confirm embryo infection, but quantification was difficult. By publishing these preliminary results, we hope that not only our group but others within the scientific community further this research towards the establishment of biomimetic and reproducible ex vivo models of persistent infection.

## 1. Introduction

Chronic wounds are described as severe non-healing wounds that constitute a significant burden to healthcare systems [1]. According to a retrospective health economics study, in the U.K. the National Health System (NHS) treated 2.2 million wounds in the year 2012/2013, with an estimated cost of GBP 5 billion. In the USA, about 6.5 million patients are affected by chronic wounds and an estimated USD 25 billion is spent annually on treating these wounds [1]. Predictably, these figures will rise with an ageing population and the increasing prevalence of long-term conditions such as diabetes [1]. Chronic wounds do not progress through the overlapping healing stages of haemostasis, inflammation, proliferation, and remodelling, as these wounds are often infected and lack the angiogenic supply for the healing process to begin [1,2]. Current treatments include surgery, dressings, drugs, and devices, among others, and often a combination of them is necessary to achieve healing [1]. Therefore, there is a clinical need for new effective treatments with the ultimate goal of regeneration rather than repair. Tissue engineering is a rapidly progressing field that offers exciting potential to find new and regenerative treatments for chronic wounds.

With the advent of tissue engineering, biomedical engineers, material scientists, and cell biologists are researching novel systems for management of these difficult wounds. The research strategies for treating chronic wounds vary from stem cell-based therapies and gene therapies to tissue-engineered biomaterial scaffolds [3,4]. The rationale for using tissue-engineered scaffolds is to control the infection, restore the angiogenic response, and allow new tissue formation to take place while providing a temporary extracellular matrix (ECM). To develop biomaterials that can treat the underlying infection and promote angiogenesis, the candidate materials need to go through rigorous in vitro and in vivo testing. In the particular case of chronic wounds, currently there is not a chronic wound model in existence that can mimic the in vivo scenario for pre-screening biomaterials. Such a model should mimic the persistent infection associated with chronic wounds. Furthermore, this model should allow testing of the angiogenesis potential of the candidate biomaterials.

The extraembryonic chorioallantoic membrane (CAM) of the chick embryo is easily accessible, highly vascularised, and presents an immunodeficient environment [5]. Therefore, it has been widely used in various aspects of research such as biology, pharmacy, oncology, and tissue regeneration [5,6]. It has also been used as an ex vivo model to study pathogenesis and the invasion capacity of different bacteria such as *Klebsiella pneumoniae*, fungi such as *Cryptococcus gatii* or *Candida albicans*, and viruses such as the fowlpox-causing viruses [7,8,9,10]. In these studies, the CAM was inoculated with the pathogen of interest, and after subsequent infection, the researchers assessed the survival and morphological features of the CAM and embryo. Thus far, only *in ovo* systems have been used. However, *ex ovo* systems may offer advantages over *in ovo* systems. Although *in ovo* CAM assays are very popular, a significant variation exists in in ovo techniques as a result of a lack of standardisation. Furthermore, in ovo systems often develop contamination from the eggshell dust, and therefore are inefficient in maintaining sterility of the embryo [6]. Recently, research into ex ovo techniques has seen the development of efficient, reproducible, and cost-effective CAM assays that are becoming increasingly popular in the fields of biomaterials and tissue engineering [6].

We are currently developing a novel ex vivo model of persistent infection using ex ovo chick embryo CAM for testing the antimicrobial potential of tissue-engineered materials followed by their subsequent angiogenesis potential. In the present preliminary study, we try to answer the initial question of whether it is possible to infect chick embryos/CAM with bacteria and maintain their survival ex ovo for up to 48 h. For this purpose, we used *Staphylococcus aureus*, one of the main types of bacteria found in the persistent infection associated with chronic wounds [11], and antibiotics (i.e., streptomycin) to maintain and/or enhance embryo survival.

## 2. Materials and Methods

### 2.1. S. aureus Strains

A clinical strain and a control antibiotic-susceptible strain (ATCC 29213) of *S. aureus* were kindly donated by The Hillingdon Hospitals NHS Foundation Trust (Uxbridge, UK). Donated *S. aureus* samples were in mannitol salt agar (MSA) plates.

### 2.2. Luria-Bertani (LB) Agar Plates

LB agar powder (17.5 g, 22700025, Thermo Fisher Scientific, Loughborough, UK) was dissolved in 500 mL of deionised water, autoclaved, and cooled down to approximately 50 °C. Working in a biosafety cabinet, the agar was poured into Petri dishes (around 25 mL of agar per dish). Plates were partially covered with the lids and allowed to cool down and dry. Finally, the lids were closed, and the plates stored with the agar side up at 4 °C until used (for a maximum of 4 weeks).

### 2.3. S. aureus Culture/Colony Isolation

The agar plate(s) were taken out of the fridge to warm up to room temperature. Using a sterile swab, a single colony of *S. aureus* from the sample MSA plate (clinical or control strain) was picked and streaked onto a fresh LB agar plate, which was incubated for up to 24 h at 37 °C. The same procedure was carried out to maintain culture of *S. aureus* in LB agar plates but transferring individual colonies from LB agar plates to fresh ones.

### 2.4. Streptomycin Sensitivity Assay

Filter paper (Whatman, WHA1001150, Sigma-Aldrich, Dorset, UK) was cut into 6 mm discs and autoclaved prior to use. Streptomycin solutions (streptomycin sulfate salt, S6501, Merck, Feltham, UK) ranging from 100 mg/mL to 5 µg/mL were prepared and stored at 4 °C under sterile conditions.

A standard EUCAST (European Committee on Antimicrobial Susceptibility Testing) disc diffusion method [12] was used as follows. Agar plates were taken out of the fridge several minutes before the procedure. Isolated colonies of *S. aureus* were picked from an LB plate (see Section 2.3) using a sterile swab, transferred into a sterile tube containing 1 mL of sterile water, and vortexed to suspend the bacteria in the water. The Optical Density (OD) at 600 nm of the prepared bacterial suspension was measured. If required, the suspension was diluted to obtain 4 × 10^8^ cfu/mL. A sterile swab was dipped in the final suspension, and excess fluid was removed and swabbed onto a fresh LB agar plate to obtain confluent bacterial growth. Bacteria were let to set for a few minutes before applying the streptomycin-soaked discs onto the plate; using sterile tweezers, filter paper discs were dipped in the required streptomycin solution, excess fluid was removed, and then the soaked discs were placed onto the plates, which were incubated for 24 h at 37 °C. Finally, the diameters of the zones of inhibition were measured using a ruler.

### 2.5. Ex Ovo Culture System

The use of chicken embryos in our study did not require ethical approval as per the guidelines of The Institutional Animal Care and Use Committee (IACUC) and the National Institutes of Health, USA, and the Animals Scientific Procedures Act (ASPA), U.K., which state that a chicken embryo that has not reached the 14th day of its gestation period would not experience pain and can therefore be used for experimentation without any ethical restrictions or prior protocol approval [6].

Fertilized chicken eggs obtained from a local producer in Middlesex (Quedgeley, UK) were kept in a specialized incubator at 38 °C and ≈45% relative humidity for 3 days, when the embryos were transferred to a shell-less culture system consisting of a glass–cling film set-up routinely used in our laboratory [6]. Briefly, Pyrex glasses of 8 cm diameter were autoclaved and filled up to three-quarters with sterile water, and then a clean cling film layer (pre-sterilised with 70% Industrial Methylated Spirit (IMS) and dried) was placed inside the glasses with the bottom of the cling film touching the water. Rubber bands secured the cling film on the glasses. After 3 days in the specialized incubator, eggs were wiped with cytosol wipes, cracked open against the sharp edge of a triangular block, and the contents were immediately transferred to the glass-cling film set up. The yolk sac and the embryo were identified and assessed for viability by looking for a beating heart. The glasses were then covered with a Petri dish, transferred to the incubator, and grown for 6 days at 38 °C and 80%–90% humidity.

### 2.6. Ex Ovo CAM Infection with S. aureus

**Figure 1:**
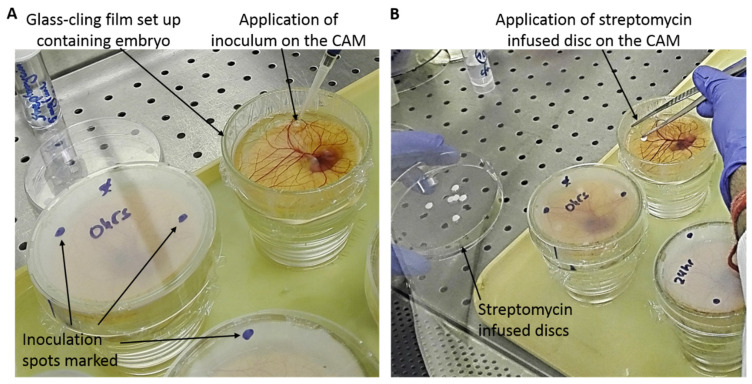
Photos taken during the experimental procedure of this study showing (**A**) application of *Staphylococcus aureus* bacterial inoculum on the chorioallantoic membrane (CAM) of a viable embryo contained in a glass–cling film set-up and inoculation spots marked on the cover Petri dish lids, and (**B**) application of streptomycin-infused 6 mm diameter filter paper discs on the CAM using sterile tweezers.

At day 9, the CAMs were infected using 2 doses of 50 µL of *S. aureus* bacterial inoculum, applied topically avoiding dispersing the inoculum very close to the embryo (Figure 1A). The inoculation spots were marked on the cover Petri dish lid (Figure 1). Control CAMs were inoculated with two doses of 50 µL of deionised water. The ex ovo cultures were covered with the corresponding Petri dish lid and incubated at 38 °C and 80%–90% humidity. Viability of the embryos was monitored daily. Macroscopic and microscopic images (using a GT vision stereo microscope, GXM-XTL3T101, GT Vision Ltd, Stansfield, UK) of the embryos were taken regularly. Allantoic fluid samples were taken either from embryos that died during the experiment or that were sacrificed if still viable. The allantoic fluid was collected (≈1 mL) at relevant time points, diluted 5–6-fold, plated, and incubated at 37 °C for 24 h to examine the presence of infection.

### 2.7. Preparation and Application of Streptomycin-Infused Discs on CAM

A solution of 5 mg/mL of streptomycin in sterile water was prepared, and 6 mm diameter filter paper discs were dipped into it. Streptomycin-infused discs (×4) were gently applied onto the CAM using sterile tweezers (Figure 1B). The ex ovo cultures were immediately covered with the corresponding Petri dish lid and incubated at 38 °C and 80%–90% humidity.

A control for streptomycin activity was run alongside the ex ovo cultures by using the standard EUCAST method described before. Four streptomycin-infused discs were applied onto the LB agar plate, which was incubated at 37 °C for up to 24 h.

## 3. Results and Discussion

### 3.1. Streptomycin Sensitivity Assay

**Figure 2:**
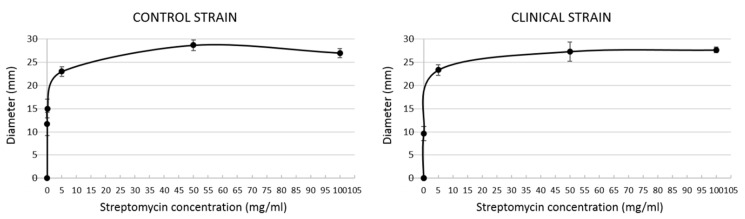
Results from the streptomycin sensitivity assay. Graphs show average ± standard deviation (*n* = 3 per streptomycin concentration).

An assay to determine the streptomycin concentration that yielded the desired level of bactericidal effect was carried out, where *S. aureus* was tested against several concentrations of streptomycin using the standard EUCAST disc diffusion method [12]. *S. aureus* from both a clinical strain and a control antibiotic susceptible strain were used. Results showed that the minimum streptomycin concentration necessary to yield a noticeable bactericidal effect on both strains tested was 5 mg/mL. Below this concentration, the bactericidal effect was faint, whereas above it the effect was hardly enhanced (Figure 2). Therefore, 5 mg/mL was chosen to carry out our study.

### 3.2. Embryo Survival after Infection of CAM in the Absence of Streptomycin

According to the literature, an inoculum of 10^8^ cfu/mL or higher would result in embryo death [7,8]. A quick test confirmed this previous finding, where our embryos died within 24 h following CAM inoculation with 10^8^–10^9^ cfu/mL inoculum (results not shown). A study by Gow et al. using an inoculum of 10^5^ cfu/mL showed embryo death within 24 h. However, in the cited study, the infecting pathogen was the fungus *Candida albicans* [10]. In our study, we lowered the bacterial load in the inoculum to 10^5^ cfu/mL for investigating embryo survival following CAM infection.

**Figure 3:**
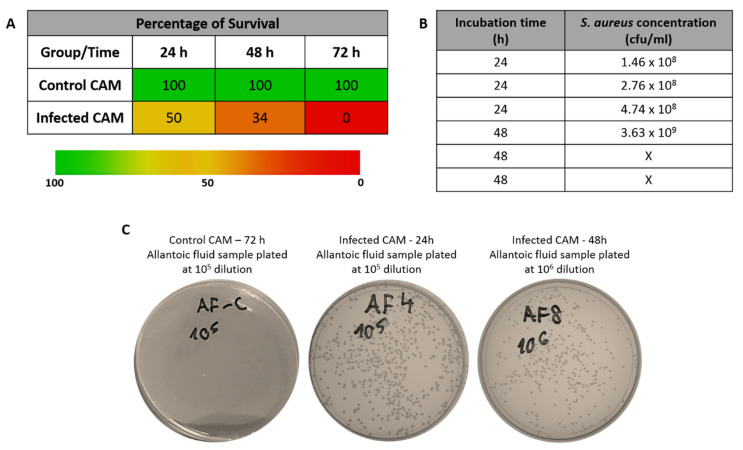
(**A**) Percentage of embryo survival after CAM infection (infected CAM) with 10^5^ cfu/mL inoculum compared to non-infected CAMs (control CAM). Results show average (*n* = 3 for control and *n* = 6 for infected CAM at the beginning of the experiment). (**B**) *S. aureus* load in allantoic fluid samples of infected CAMs. X means that there were too many colonies and a count could not be performed. (**C**) Representative images of Luria-Bertani (LB) plates showing *S. aureus* colony formation after plating samples of the allantoic fluid from control and infected CAMs.

Results showed that embryo survival decreased to 50% 24 h after inoculation and continued to decrease following another 24 h of incubation (Figure 3A). By day 3 (72 h), infected embryos were dead. Our control embryos (non-inoculated CAM) survived over the 72 h experiment (Figure 3A). On examining the allantoic fluid, the presence of bacterial colonies was observed in infected embryos, suggesting the presence of infection within the CAM as well (Figure 3B,C).

These results suggest that it is possible to infect chick embryos/CAM with *S. aureus* (10^5^ cfu/mL inoculum) and maintain their survival for up to 48 h. However, the percentage of embryo survival under these conditions is approximately 30%. Therefore, the next question to answer is whether survival rate could be increased with the use of antibiotics, the aminoglycoside streptomycin in our study.

### 3.3. Embryo Survival after Infection of CAM in the Presence of Streptomycin

**Figure 4:**
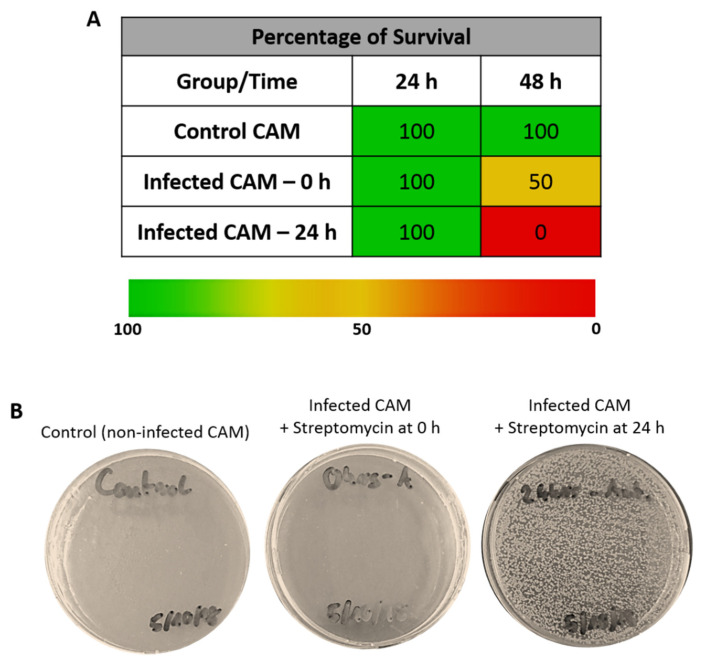
(**A**) Percentage of embryo survival after CAM infection (infected CAM) with 10^5^ cfu/mL inoculum compared to non-infected CAMs (control CAM). Streptomycin was applied either at the time of inoculation (0 h) or 24 h after inoculation (24 h). Results show average (*n* = 2 per group). (**B**) Representative images of LB plates showing *S. aureus* colony formation after plating allantoic fluid samples (10^5^ dilution). No colonies were observed in the control group plates. As can be observed, there were too many colonies to perform a count and subsequent quantification, which could only be done using a higher dilution.

An increase in embryo survival was observed in the presence of the antibiotic streptomycin when applied at the time of inoculation (0 h) (Figure 4A). On injecting the CAMs with the antibiotic 24 h after inoculation, survival increased after 24 h incubation; however, after further 24 h of incubation, the embryos died. Examination of the allantoic fluid showed clear presence of infection in the embryos/CAM when the antibiotic was applied 24 h after inoculation, whereas no infection could be seen in the control group or when streptomycin was applied at the time of inoculation (Figure 4B).

These results suggest that embryo survival depends on the inoculum dose, the use of antibiotics, and the time of their application on the infected CAM. Further work on this project intends to look into a higher range for these variables to find optimum conditions. Nevertheless, the answer to the initial question of whether it is possible to infect chick embryos/CAM with bacteria and maintain their survival for up to 48 h was affirmative, which sets the ground for developing an ex vivo model of persistent infection.

### 3.4. CAM Morphology of Infected Embryos

Another aim of this study was to investigate CAM morphology of infected embryos to understand how infection with *S. aureus* affects the CAM blood vessel network, which could have an effect when studying the angiogenic potential of candidate materials. The CAM is highly vascularised with both mature vessels and capillaries and is easily accessible for orthotopic implantation of biomaterials without initiating an embryo immune reaction [6]. If this network of vessels and capillaries was badly disrupted due to infection, testing of biomaterials on it would not be possible.

**Figure 5:**
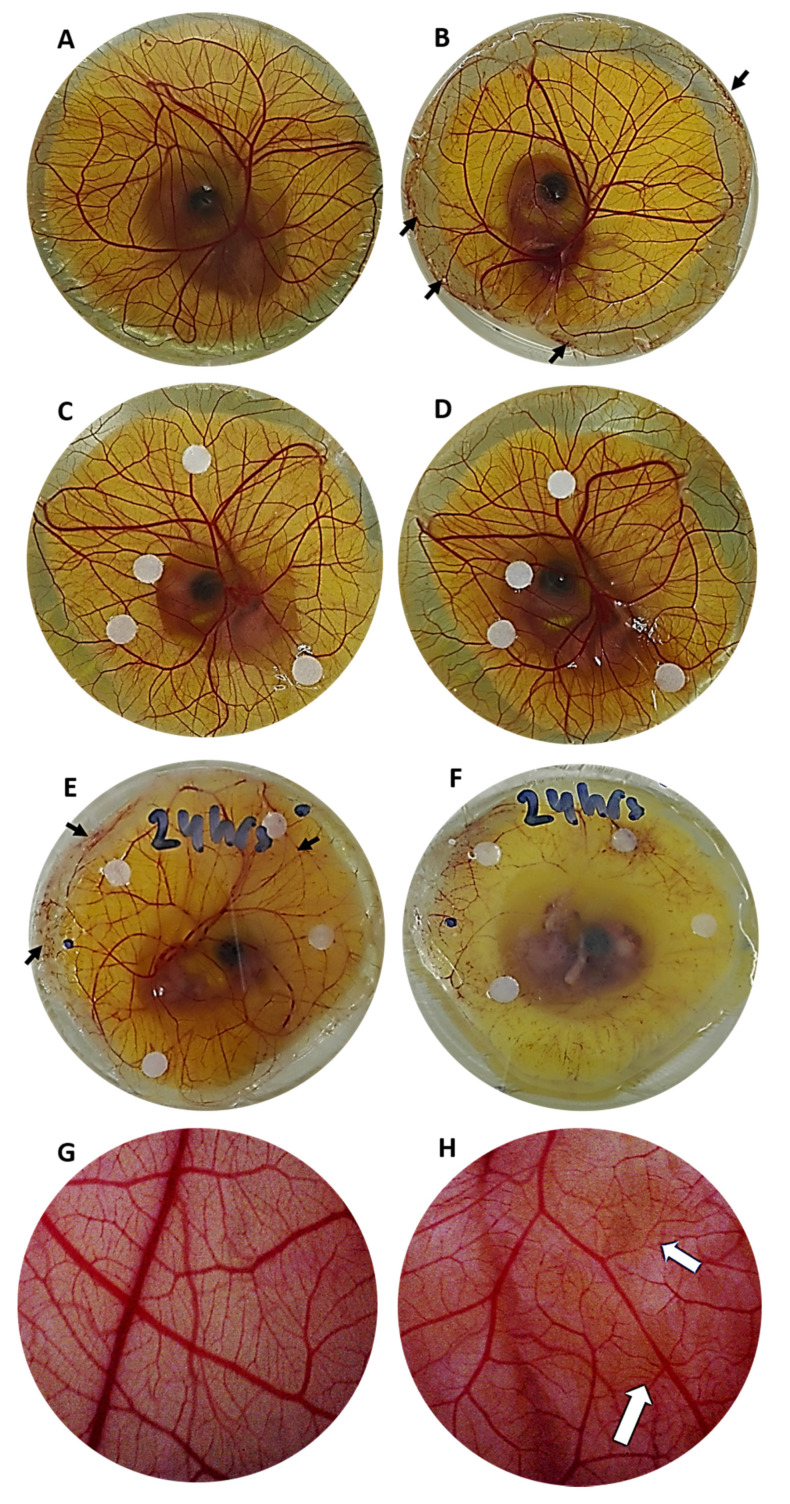
Macroscopic images of the CAM with and without bacterial infection, where black arrows point at vessel damage due to the bacterial invasion. (**A**) Non-infected CAM incubated for 48 h. (**B**) CAM infected with 10^5^ bacterial inoculum and incubated for 48 h. (**C**) CAM infected with 10^5^ bacterial inoculum with antibiotic applied at the time of infection (0 h) and incubated for 24 h. (**D**) Same embryo as C after 48 h incubation. (**E**) CAM infected with 10^5^ bacterial inoculum with antibiotic applied 24 h post-infection and incubated for 24 h. (**F**) Same embryo as (**E**) after 48 h incubation (embryo is dead and network of blood vessels is completely destroyed). (**G**) Stereomicroscopy image of non-infected CAM at 24 h showing a mostly clear background and well-defined blood vessels. (**H**) Stereomicroscopy image of infected CAM at 24 h post-infection showing a cloudy background (white arrows point at yellowish cloudy spots) and in some areas blood vessels appear less well-defined and fainter than in non-infected CAM. Please note that with the purpose of protecting the live ex vivo cultures, photos were taken with the Petri dish lid on, but the lid might have been taken off if the embryos were already dead or sacrificed. Additionally, the lid might have been moved (without opening) to capture the morphological changes in the CAM/embryo.

Non-infected CAMs showed a well-defined network of blood vessels with no obvious pathological features (Figure 5A,G). On the other hand, CAMs with signs of infection showed various degrees of blood vessel damage, which was mainly observed on the periphery of the CAM, as well as throughout the network (Figure 5B,E). In some instances, the blood vessel network appeared quite disrupted, making it not suitable for further biomaterial testing (Figure 5E). However, some infected embryos showed minimal blood vessel network disruption (Figure 5B). Microscopic analysis showed a cloudy background for infected CAMs (Figure 5H) compared to non-infected CAMs (Figure 5G). Application of streptomycin at the time of inoculation seemed to clear infection because no evident signs of it could be observed for the majority of these CAMs (Figure 5C,D) compared to applying the antibiotic 24 h after inoculation (Figure 5E). When embryo death occurred, the CAM blood vessel network appeared completely destroyed (Figure 5F).

These results suggest that under certain conditions it is possible to infect CAMs with *S. aureus* and keep the embryo viable while maintaining the network of CAM blood vessels with minimal disruption. In the future, it would be useful to investigate the way in which to quantify blood vessel damage to establish a threshold of viability for biomaterial testing.

We acknowledge that the system presented here is, at the moment, fragile, where we only managed to keep a low percentage of infected embryos alive for up to 48 h. We also acknowledge variation of outcomes and reproducibility. The CAM model in itself is a highly biological variable, even in non-infected eggs [6]. Therefore, the differences observed in our study may stem from this intrinsic feature of the CAM model, rather than inappropriate methodology. Further experiments with increased biological replicates may improve reproducibility.

It is worth mentioning that extraction of allantoic fluid was technically difficult and invasive to the embryo. Although information on the dynamics of infection spread would be useful, manipulation of the embryo during the experiment could introduce additional contamination and stress, increasing the chances of death due to the experimental procedure versus the infection itself. However, this can be resolved by extracting the allantoic fluid after sacrificing the embryo, as we did in our study, at given time intervals. The analysis of allantoic fluid in this preliminary study was largely exploratory and we could not anticipate the final bacterial concentration in the embryo because no reports of similar experiments exist. In future work, we do intend to take into account this study’s observations when quantifying bacterial burden. Notwithstanding, the results presented still allow comparison of bacterial burden between CAMs with different survival outcomes.

In addition, more research and optimisation are needed to test a wider range of conditions, such as the ones mentioned earlier (i.e., inoculum dose, the use of antibiotics, and the time of their application on the infected CAM), in order to find the optimum conditions to achieve consistent and reproducible viability of infected embryos. Nevertheless, this system is more representative of the biological situation in chronic wounds where living tissues are ridden with bacterial infection. Moreover, it is very cost-effective.

## 4. Conclusions

The main conclusions from this study are (1) it was possible to infect chicken embryos with *S. aureus*, one of the main types of bacteria found in the persistent infection associated with chronic wounds, and maintain the embryos’ survival for up to 48 h; (2) survival of the embryos varied with the dose of bacteria inoculum and with the use and time of application after infection of an antibiotic (streptomycin in this study); (3) in infected yet viable embryos, the blood vessels network of the CAM was maintained with minimal disruption; and (4) microbiological tests can confirm infection of the embryo, but quantification is difficult.

In summary, we report here preliminary data towards the development of a live ex vivo model of persistent infection that can be used for pre-screening biomaterials intended for treating chronic wounds for their antimicrobial and angiogenic potential. This model is relatively simple, quick, and low-cost, and mimics the in vivo situation more closely than traditionally used antimicrobial tests using agar plates and dilution assays. In addition, keeping in accordance with the principles of the National Centre for the Replacement Refinement and Reduction of Animals in Research (NC3R’s), this model does not require administrative procedures for obtaining ethics committee approval for animal experimentation. Nevertheless, we do acknowledge that further work is still needed towards further optimisation to study the interaction of biomaterials with two different strains of bacteria commonly found in chronic wounds, in order to assess their antimicrobial and angiogenic properties. By publishing these preliminary results, we hope that not only our group but others within the scientific community further this research towards the establishment of biomimetic and reproducible ex vivo models of persistent infection. Such models would not only be useful in the research of treatments for chronic wounds, but also in those applications where infection is an important factor to take into account when investigating new therapies.
